# Light-Activatable, Cell-Type Specific Labeling of the Nascent Proteome

**DOI:** 10.1021/acschemneuro.4c00274

**Published:** 2024-09-23

**Authors:** H. T. Evans, T. Ko, M. M. Oliveira, A. Yu, S. V. Kalavai, E. N. Golhan, A. Polavarapu, E. Balamoti, V. Wu, E. Klann, D. Trauner

**Affiliations:** † Center for Neural Science, New York University, New York, New York 10003, United States; ‡ Department of Chemistry, University of Pennsylvania, Philadelphia, Pennsylvania 19104, United States

**Keywords:** mRNA translation, photopharmacology, light-inducible, cell-type specific, de novo proteome, photocage

## Abstract

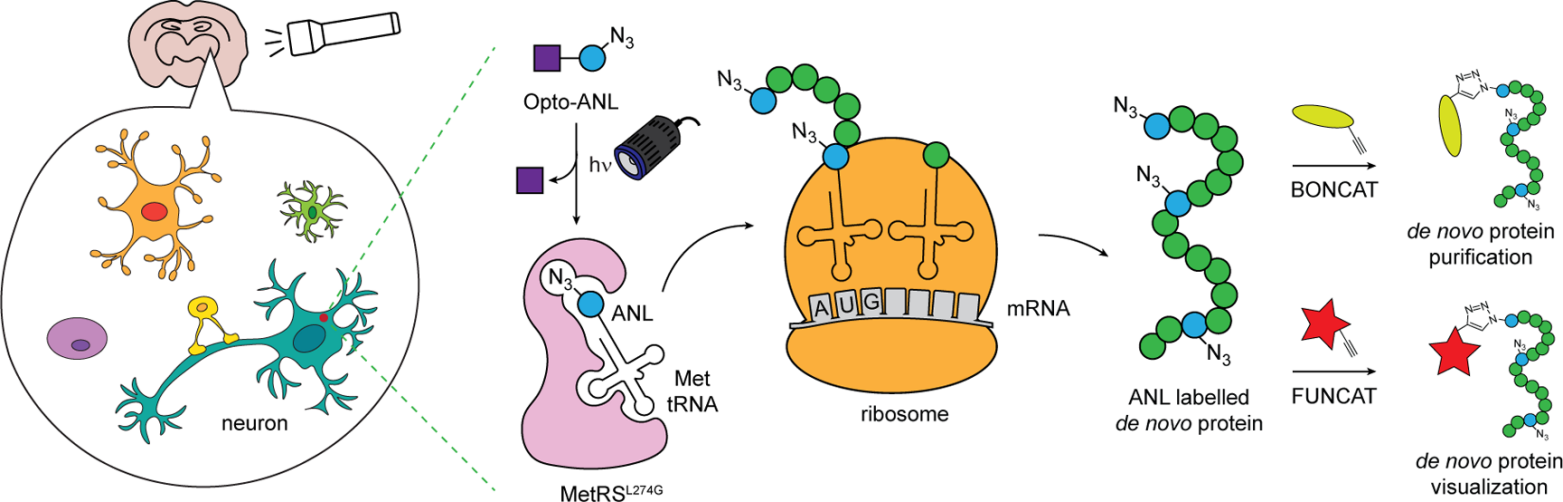

Elucidating the mechanisms by which protein synthesis contributes to complex biological processes has remained a challenging endeavor. This is particularly true in the field of neuroscience, where multiple, tightly regulated periods of new protein synthesis in different cell-types are thought to facilitate intricate neurological functions, such as memory formation. Current methods for labeling the *de novo* proteome have lacked the spatial and temporal resolution to accurately discriminate these overlapping and often competing windows of mRNA translation. To address this technological limitation, here we describe a novel, light-inducible specific method for labeling newly synthesized proteins within a targeted cell-type.By developing Opto-ANL, a photocaged version of the nonendogenous amino acid azidonorleucine (ANL), we can selectively label newly synthesized proteins in specific cell-types through the targeted expression of a mutant methionyl-tRNA synthetase (L274G-MetRS). We demonstrate that Opto-ANL can be rapidly uncaged by UV light treatment in both cell culture and mouse brain slices, with Opto-ANL labeled proteins being able to be visualized via fluorescent noncanonical amino acid tagging. We also reveal that pretreatment with Opto-ANL not only allows for the period of *de novo* proteomic labeling to be tightly controlled, but also improves labeling efficiency compared to regular ANL. To demonstrate the potential applications of this novel technique, we use Opto-ANL to detect insulin-induced increases in protein synthesis and to label the excitatory neuronal *de novo* proteome in mouse brain slices. We believe that this application of photopharmacology will allow researchers to generate novel insights into how the translational landscape is altered across cell-types during complex neurological phenomena such as memory formation.

## Introduction

The translation of mRNA into newly synthesized proteins, both in the cell body and at the synapse, allows neurons to dynamically control the levels of proteins throughout the cell. mRNA translation is a highly regulated process and is vital in allowing neurons to respond to both physiological and pathological signals. In the central nervous system, protein synthesis is studied primarily in the context of long-term memory.

Protein synthesis was first demonstrated to be important in memory in a series of seminal studies which revealed that the administration of protein synthesis inhibitors prevents the consolidation of new long-term memories.^1^ Building upon this, researchers have been able to identify specific brain regions, such as the hippocampus and amygdala, in which protein synthesis is required for memory consolidation, reconsolidation, and extinction in various behavioral paradigms.^2,3^ Protein synthesis has also been shown to be required to facilitate the molecular changes in synaptic strength which are thought to underlie memory, including long-term potentiation and long-term depression.^4−6^ Recent technological advancements have allowed researchers to explore the role of protein synthesis in memory to a previously unobtainable degree of specificity. For example, using a cell-type specific, chemogenetic approach, researchers have been able to identify neuronal subpopulations in which translation initiation is required for the formation of new cued-threat conditioned memories.^7,8^

**Figure 1:**
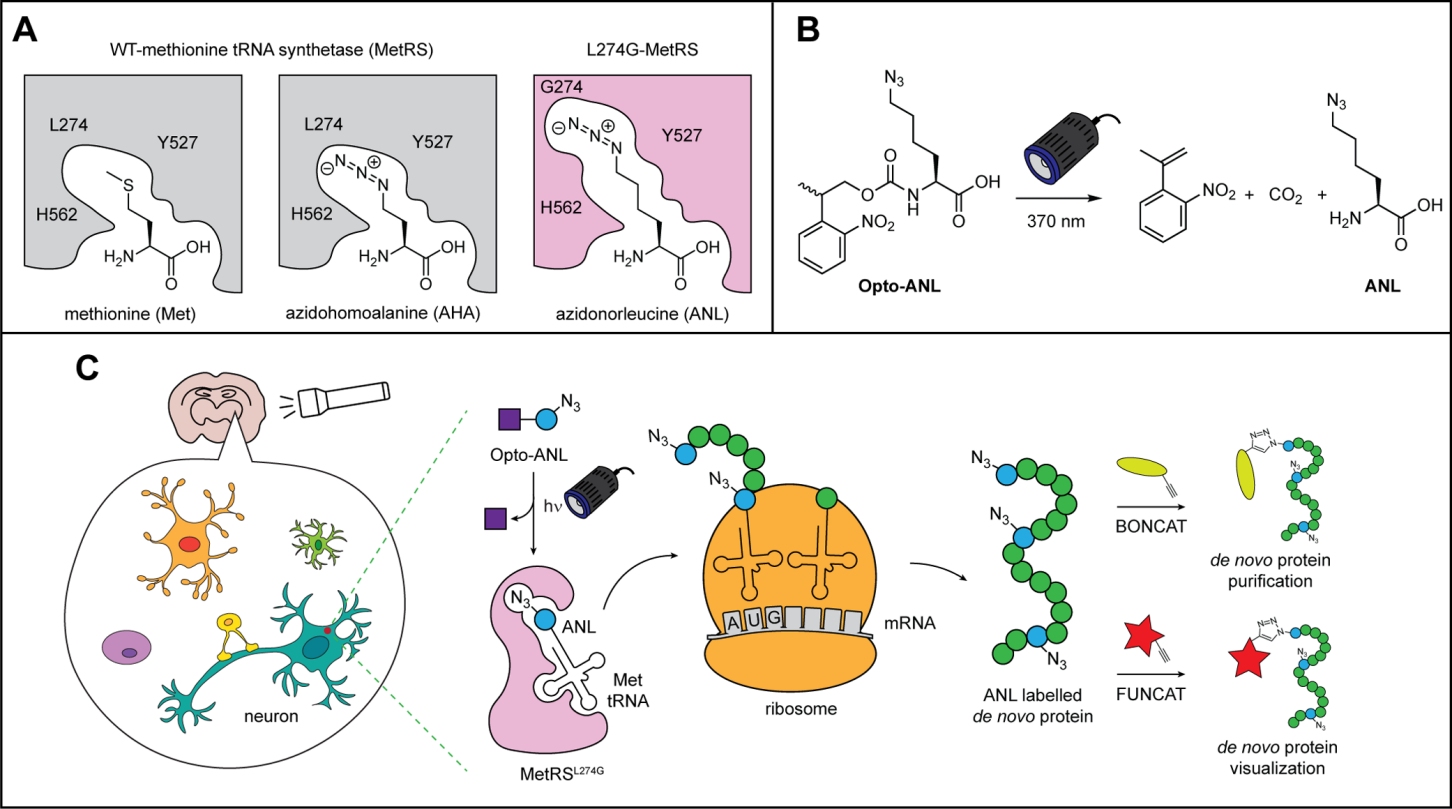
(A) Schematic depicting the binding of methionine and its analogues to wild-type and mutant L274G MetRS. (B) Opto-ANL can undergo uncaging upon UV light irradiation to release ANL. (C) Scheme depicting the cell-type specific incorporation of Opto-ANL upon light irradiation in brain slices. ANL labeled proteins synthesized in cells expressing L274G-MetRS can be purified or visualized via BONCAT or FUNCAT, respectively.

Given the importance of protein synthesis in memory, identifying which proteins that are synthesized with cell-type and temporal specificity during the formation of new long-term memories would provide invaluable insights into the molecular mechanisms that are involved in this process. The current gold-standard for studying the *de novo* proteome involves labeling newly synthesized proteins with noncanonical amino acids (NCAAs), the most widely used of which is azidohomoalanine (AHA).^9^ An azide-bearing surrogate of methionine, AHA is recognized the endogenous methionine tRNA synthetase (MetRS) and loaded onto both the initiator and elongator methionine tRNA before being incorporated into the nascent polypeptide chain.^10^ The nonendogenous azide group present in these NCAAs then allows for labeled proteins to be covalently bonded to an alkyne-bearing tag, enabling either their visualization via fluorescent noncanonical amino acid tagging (FUNCAT) or their purification and subsequent analysis via bio-orthogonal amino acid tagging (BONCAT) (Figure 1C).^11^

AHA labeling has been used to study protein synthesis in the central nervous system in a wide variety of contexts, including identifying impairments in mRNA translation in models of neurodegeneration,^12−14^ measuring protein degradation rates,^15^ examining microglial protein synthesis,^16^ identifying synaptic changes in the nascent proteome during homeostatic scaling,^17^ and detecting changes in the *de novo* proteome following a training in a spatial memory paradigm.^18^ Despite its widespread adoption, AHA labeling does have some limitations, primarily the lack of cell-type specificity. The nascent proteomes of various subpopulations of neurons, such as excitatory and inhibitory neurons, have been shown to differ greatly, with these *de novo* proteomic differences being even more pronounced between neurons and glial cells.^19^ Furthermore, these different cell types likely play different and even contradictory roles in facilitating long-term memory formation. As AHA is recognized by the endogenous translational machinery, its incorporation cannot be restricted to one particular cell type and, as such, AHA labeling is ill-suited to exploring how the *de novo* proteomes of specific cell types are altered during long-term memory formation.

To explore cell-type specific changes in protein synthesis, researchers have instead turned to other NCAAs which are not recognized by the endogenous translational machinery, such as azidonorleucine (ANL).^20^ Although ANL is also a methionine surrogate, unlike AHA, it is not recognized by the endogenous MetRS, instead requiring the expression of a mutant methionyl-tRNA synthetase, such as L274G-MetRS, for its aminoacylation to the methionine tRNAs (Figure 1A).^21^ As a result of this, it is possible to restrict ANL labeling of the *de novo* proteome to a specific cell type by genetically restricting the expression of the mutant tRNA synthetase to said cell type. ANL labeling has been previously used to explore the *de novo* proteomic difference between neuronal subpopulations after environmental enrichment,^22,23^ and to identify changes in the nascent proteome of hippocampal excitatory neurons following training in spatial long-term memory paradigm.^18^

One factor that has prevented NCAA labeling from being more broadly used to study long-term memory-induced protein synthesis is the relatively extended labeling periods. Long-term memory is thought to be dependent on multiple, temporally distinct, windows of protein synthesis which occur in the first 24 h following learning.^24^ Although NCAAs like ANL and AHA can be incorporated into the nascent proteome in as little as 15 min in cells,^25^ achieving sufficient labeling in vivo in rodents takes substantially longer, with previous studies labeling the *de novo* proteome over at least 16 h.^13,18,22^ As a result, ANL labeling is currently unable to distinguish between the multiple windows of memory-induced protein synthesis.

To overcome the limitations of ANL labeling, we sought to create a photoactivatable version of ANL that could be delivered to neurons before being activated at a later time point, and as a result increase the temporal and spatial control of ANL labeling. This compound, termed Opto-ANL, was designed to rapidly release ANL upon mild UV light irradiation and proteomically label cells expressing L274G-MetRS (Figure 1B,C). Pretreatment and photolabeling with Opto-ANL shows rapid and increased proteomic labeling when compared to ANL itself, allowing detection, with greater resolution, of proteomic fluctuations caused by insulin. These features make Opto-ANL a powerful tool for light-activatable and cell-type specific *de novo* proteomic labeling and a promising approach to identifying changes in protein expression in short time windows.

## Results

### Design and Synthesis of Opto-ANL

Numerous photocaged amino acids have been previously reported, with the majority of these molecules harboring a photocage at their side-chains.^26^ When designing Opto-ANL, we reasoned that caging the N-terminus of ANL would prevent its aminoacylation onto methionine tRNAs by L274G-MetRS, and its subsequent incorporation into nascent peptide chains. Moreover, an ester photocage linkage in the C-terminus could be susceptible to background hydrolysis from esterases. Previously a 2,5-dioxopyrrolidin-1-yl (2-(2-nitrophenyl)propyl) carbonate (NPPOC) photocage was utilized to prevent translation of the NCAA AHA.^27^ We sought to apply this strategy to ANL as NPPOC photocages show short photolysis times upon mild UVa irradiation due to their relatively high quantum yield when compared to other more commonly used 2-nitrobenzyl photocages.^28^ Furthermore, the presence of a β-methyl group promotes rapid α-hydrogen abstraction by the photoexcited nitro group through an alternative photocleavage mechanism, resulting in an α-methylstyrene byproduct rather than a toxic nitroso byproduct of 2-nitrobenzyl photocages.^29,30^ We thus hypothesized that the use of the NPPOC photocage would allow for more rapid release of ANL, while minimizing the harmful effects of prolonged UV light exposure and cytotoxic photocage byproducts.

**Figure 2:**
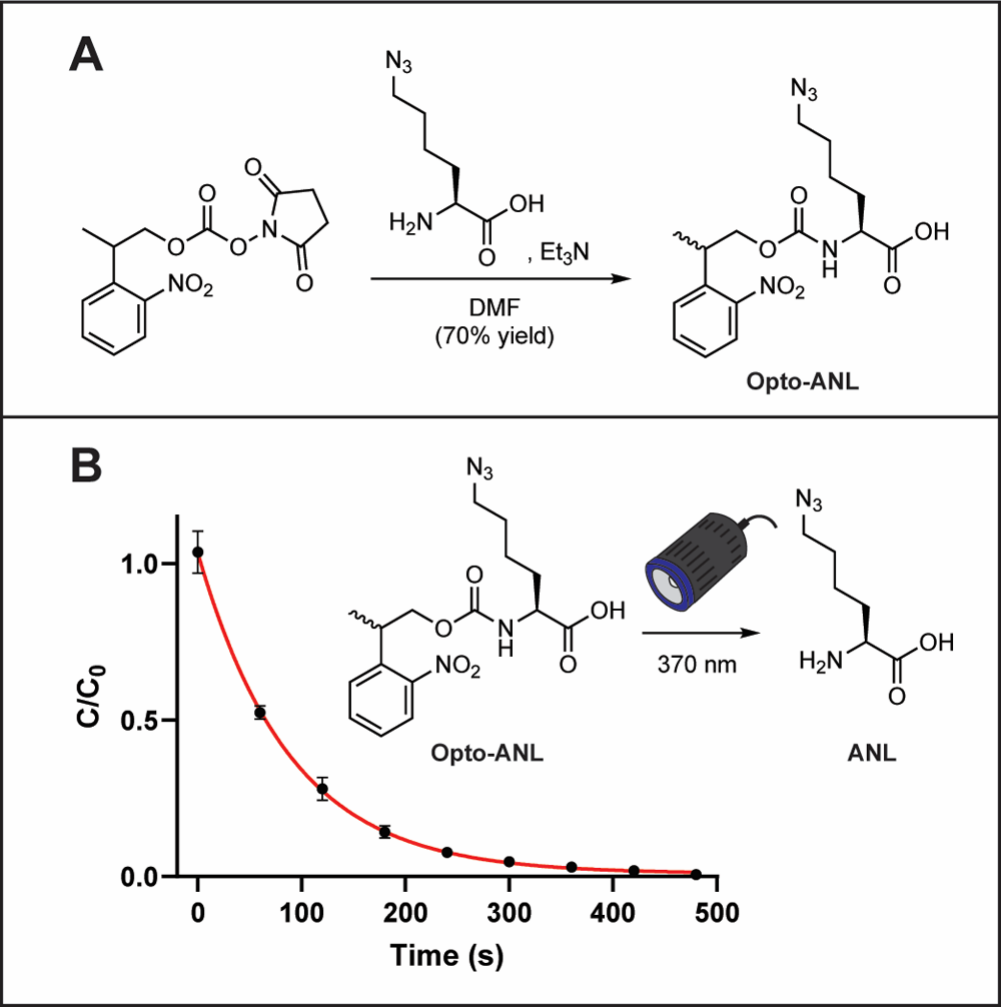
(A) Synthesis of Opto-ANL (B) kinetic analysis of Opto-ANL photolysis upon light irradiation with 370 nm light (10 mW cm^2^).

Synthesis of Opto-ANL was achieved through the condensation of commercially available ANL and the active ester of NPPOC. The active ester was synthesized through an esterification of 2-(2-nitrophenyl)propan-1-ol with DSC.^31^ (Figure 2A) To evaluate the uncaging kinetics of Opto-ANL, we monitored its photolysis by LCMS and confirmed the results by ^1^H NMR spectroscopy. Upon mild UV light (370 nm, 10 mW/cm^2^), the NPOCC photocaged amino acid could undergo almost complete photolysis within 5 min of irradiation (Figures 2B and S1 and S2). These uncaging kinetics are comparable to that observed in other uses of NPOCC as a photocage. We reasoned that this rapid release would provide us with excellent time resolution for proteomic labeling in biological settings.

### Opto-ANL Enables Light-Triggered and Cell-Type Specific Proteomic Labeling

In order to validate that Opto-ANL allows for the light-activatable, cell-type specific labeling of the *de novo* proteome, we first sought to test this labeling technique in a simple cell system. HEK293 cells were transfected with a plasmid expressing mCherry-tagged L274G-MetRS after which cells were treated with 1 mM of Opto-ANL and then irradiated for 5 or 15 min using light irradiation from our cell DISCO system (370 nm, 4.0–6.0 mW).^32^ After a labeling period of 4 h, newly synthesized proteins were visualized using FUNCAT. We observed a light dependent effect in the proteomic labeling with 15 min of irradiation providing a similar level of proteomic labeling to our ANL control, with partial labeling being observed after 5 min of irradiation (Figure S3).

**Figure 3:**
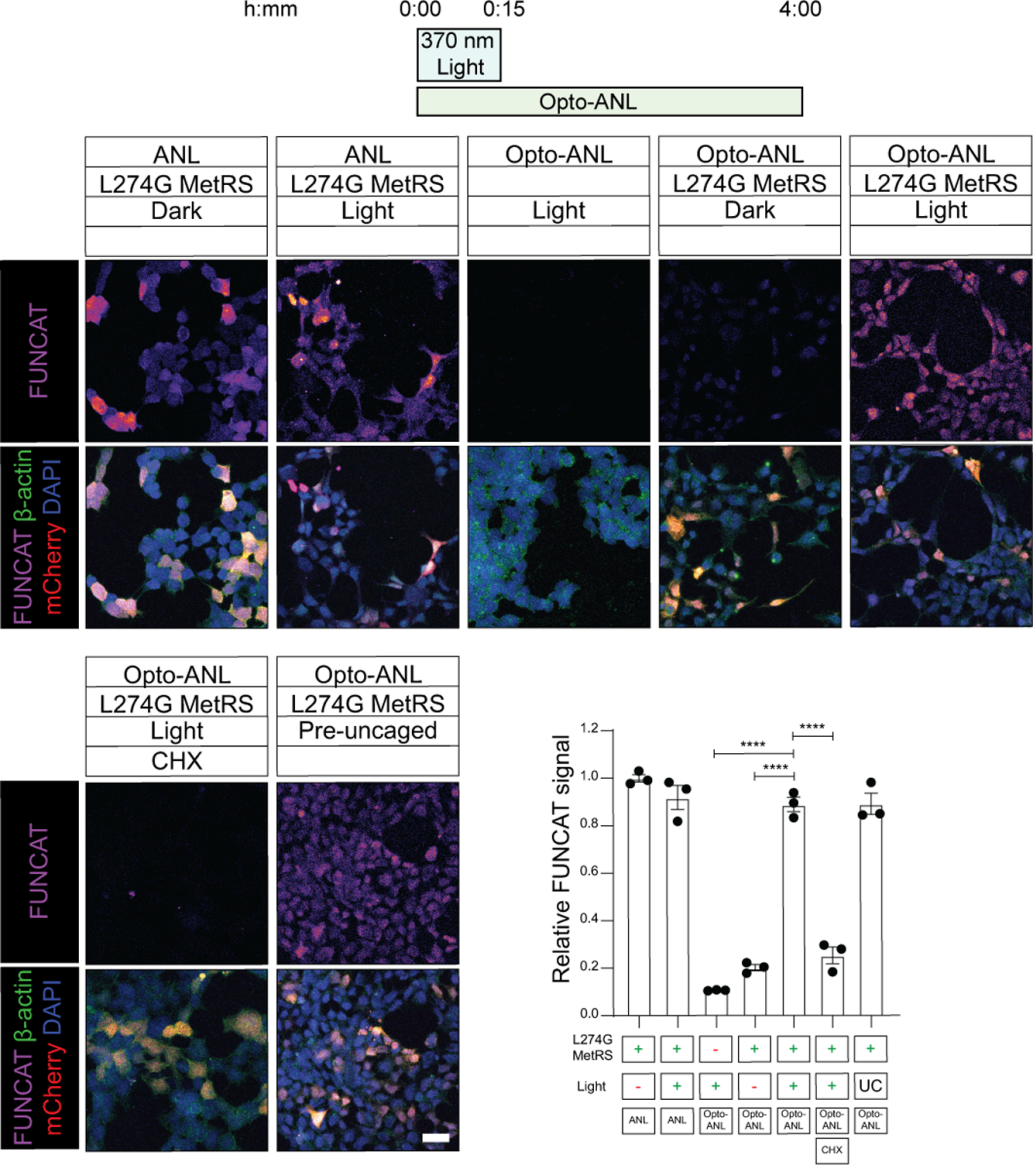
Opto-ANL enables light-activatable, L274G-MetRS dependent labeling of newly synthesized proteins. HEK293 cells were transfected with L274G-MetRS, before being treated with Opto-ANL and then irradiated with mild UV light for 15 min. This treatment resulted in similar levels of FUNCAT labeling as ANL, ANL + light, and Opto-ANL uncaged prior to treatment, suggesting that our irradiation paradigm is sufficient to induce complete uncaging of Opto-ANL, while not altering global protein synthesis levels. FUNCAT signal was ablated in the absence of light or L274G-MetRS, and is also reduced in the presence of the protein synthesis inhibitor, CHX (one-way ANOVA, Tukey’s MCT, *n* = 3 experiments, 4 technical replicates per experiment, **** = *p* ≤ 0.0001, error bars = S.E.M).

Next, we wanted to ensure that Opto-ANL labeling was dependent upon expression of a mutant-tRNA synthetase. To this end, we compared cells transfected with L274G-MetRS to untransfected cells. Cells were treated with 1 mM Opto-ANL before being irradiated for 15 min. The resulting fluorescent labeling with FUNCAT showed almost no signal for the untransfected group when compared to the cells expressing L274G-MetRS (Figure 3). This result indicates that Opto-ANL is incorporated into protein only in cells expressing a mutant tRNA synthetase, enabling this technique to be used in a cell-type specific manner. We found that cells treated with Opto-ANL and our mild-UV uncaging protocol showed similar levels of FUNCAT signal to cells treated with Opto-ANL which can be uncaged prior to be added to cells using a powerful UV lamp (Figure 3), demonstrating that our uncaging protocol results in rapid and efficient uncaging of Opto-ANL. Additionally, we confirmed that the FUNCAT signal arising from Opto-ANL treatment is protein synthesis-dependent, as this signal was ablated by pretreatment with the protein synthesis inhibitor, cycloheximide (CHX) (Figure 3). We also were able to confirm that our light irradiation protocol showed no significant effect on protein synthesis (Figure 3), and had no observable cytotoxic effects (Figure S4). Lastly, we utilized FUNCAT followed by Western blot analysis to validate our findings that Opto-ANL can be used to label the *de novo* proteome in a light and L274G-MetRS dependent manner (Figure S5).

### Opto-ANL can Detect Changes in mRNA Translation with High Temporal Resolution

**Figure 4:**
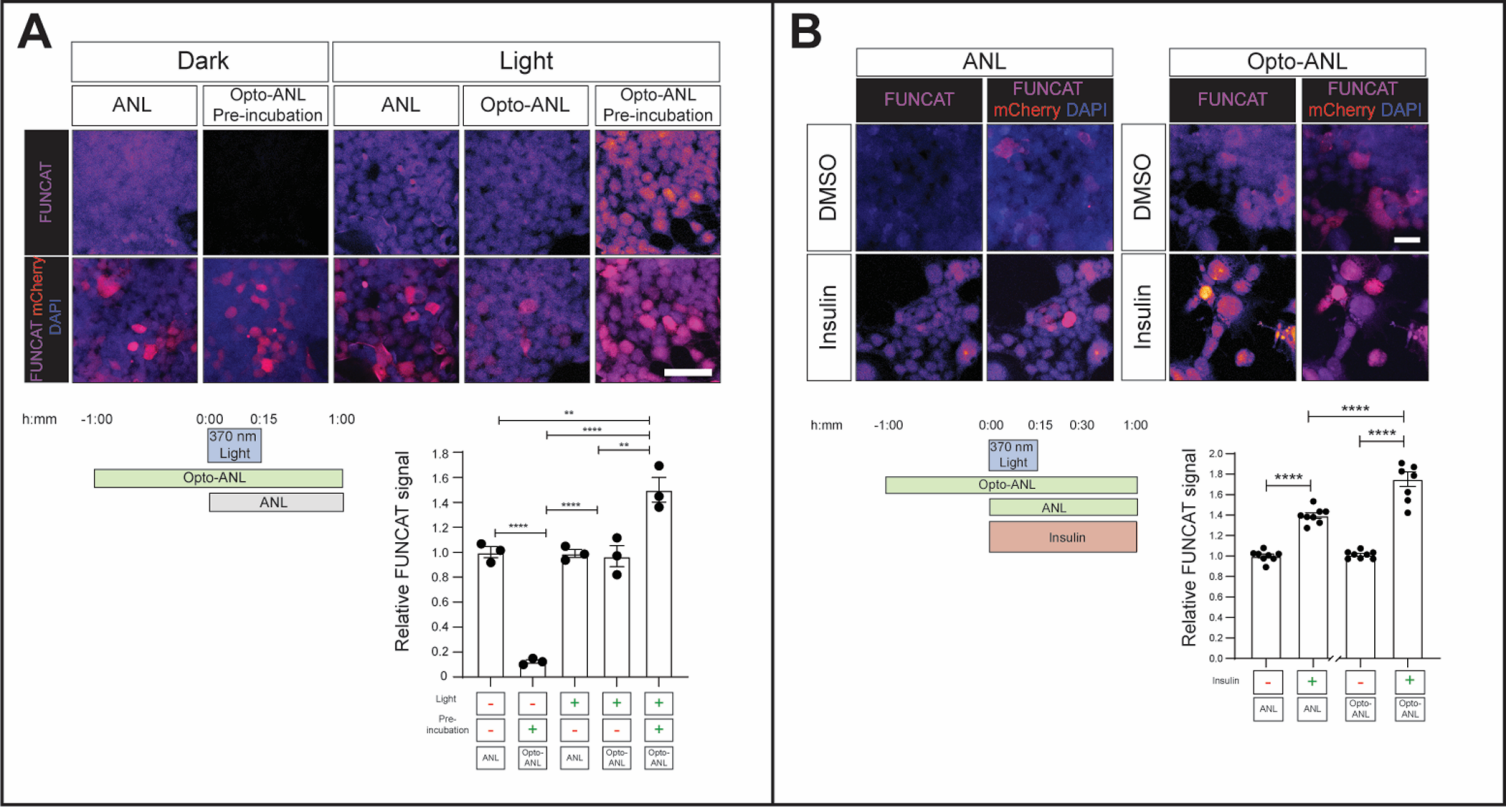
Preincubation with Opto-ANL enables increased *de novo* proteomic labeling for short time windows. (A) Cells preincubated in the dark with Opto-ANL for 1 h showed significantly increased FUNCAT labeling compared to cells which were not preincubated with either ANL or Opto-ANL (one-way ANOVA, Tukey’s MCT, *n* = 3 experiments, 4 technical replicates per experiment, ** = *p* ≤ 0.01 **** = *p* ≤ 0.0001, error bars = S.E.M). (B) While both Opto-ANL and ANL labeling can detect insulin-induced increases in protein synthesis, preincubation with Opto-ANL increased the size of the difference detected (one-way ANOVA, Tukey’s MCT, *n* = 8 experiments, 3 technical replicates per experiment, FUNCAT signal normalized to the DMSO control within each group, error bars = S.E.M).

As Opto-ANL is inactive before its uncaging, we reasoned that incubating cells with Opto-ANL prior to uncaging would allow for greater proteomic labeling. To test for this, we pretreated HEK293 cells expressing L274G-MetRS with 1 mM Opto-ANL for 1 h before uncaging. We hypothesized that this preincubation paradigm would increase the temporal resolution of Opto-ANL labeling and therefore elected to only label proteins for an additional 45 min in the dark following 15 min of UV irradiation. FUNCAT analysis revealed that preincubating with Opto-ANL increased *de novo* protein labeling by approximately 50% when compared to both ANL and Opto-ANL without preincubation (Figure 4A).

We proceeded to determine whether Opto-ANL could be used to measure changes in protein synthesis triggered by biological stimulus. Insulin is known to rapidly increase protein synthesis by activating components of the translational machinery.^33^ Using the previous pretreatment protocol with Opto-ANL, protein production in transfected HEK293 cells was stimulated by addition of insulin, and at the same time cells were irradiated with 370 nm light for 15 min. Newly synthesized proteins then were labeled for a further 45 min before FUNCAT was used to measure protein synthesis (Figure 4B). This analysis demonstrated that not only is our Opto-ANL labeling paradigm able to detect insulin-induced increases in mRNA translation but able to do so with a greater sensitivity than ANL labeling (Figure 4B).

### Opto-ANL can Distinguish between Different Cell-Types in Mice Brain Slices

**Figure 5:**
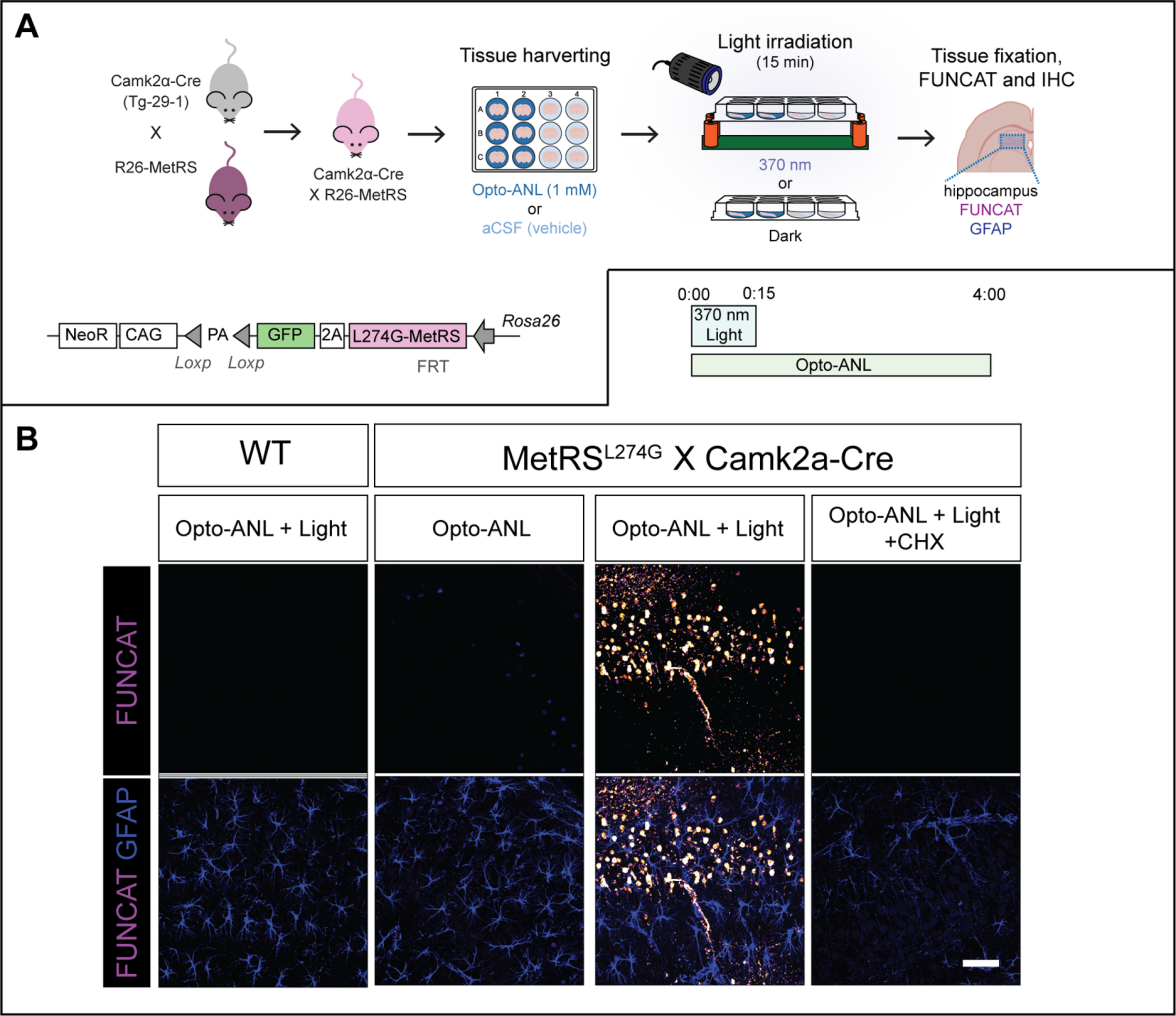
Opto-ANL enables light-activatable labeling of hippocampal neuronal *de novo* proteomic in mice. (A) In R26-MetRS mice, the gene encoding L274G-MetRS, downstream from a floxed-stop cassette, is inserted in the *Rosa26* locus. In the presence of Cre-recombinase, this stop cassette is removed, allowing for the expression of L274G-MetRS. R26-MetRS mice were crossed to Camk2a-Cre mice, which express Cre in hippocampal excitatory neurons. Live hippocampal slices taken from these mice were treated with Opto-ANL and irradiated with 370 nm light for 15 min (B). Opto-ANL enabled the light activatable labeling of newly synthesized proteins specifically in CA1 hippocampal pyramidal neurons, with no FUNCAT signal being observed in other cell-types, such as astrocytes. FUNCAT staining was ablated in WT mice, by the addition of CHX, and in the absence of UV irradiation, confirming that Opto-ANL enables the cell-type specific, light-activatable labeling of newly synthesized in mouse brain slices.

Having established the benefits of our labeling technique in a simple cell system, we next sought to leverage Opto-ANL to label neuronal protein synthesis in brain tissue. To achieve this, we utilized R26-MetRS mice which express L274G-MetRS in a Cre recombinase-dependent manner (Figure 5A).^22^ These mice were crossed with the Camk2a-Cre (T29–1) which express Cre in excitatory neurons throughout the forebrain, including the CA1 pyramidal neurons of the hippocampus.^34^ Hippocampal sections from these mice, as well as from wild-type controls then were cultured and incubated with Opto-ANL before being irradiated with UV light of 15 min, with newly synthesized proteins then being labeled for 4 h. FUNCAT and immunohistochemical analysis revealed that in L274G-MetRS X Camk2a-Cre brain tissue, Opto-ANL was able to label newly synthesized proteins specifically in CA1 hippocampal pyramidal neurons, with no FUNCAT signal being observed in other cell-types, such as astrocytes (Figure 5B). FUNCAT signal was not detected in wild-type mice, in the absence of UV irradiation, or in the presence of a protein synthesis inhibitor, confirming that Opto-ANL enables the cell-type specific, light-activatable labeling of newly synthesized in the rodent brain.

## Discussion

Numerous complex processes within the central nervous system are dependent upon the dynamic and intricate regulation of mRNA translation and as such identifying changes in proteomic dynamics is crucial for elucidating the basic cellular mechanisms which govern these neurological functions. Given that many of these processes, such as long-term memory formation, are thought to be dependent upon multiple waves of protein synthesis in a variety of different cell types,^19,24^ there exists a need for tools that can observe these discrete changes.

In this work, we introduce Opto-ANL as a sophisticated tool capable of cell-type specific *de novo* proteomic labeling with the added temporal precision of light activation. The primary advantage of this technique is that it allows for shorter and more intense *de novo* proteomic labeling of specific cell-types when compared to other labeling techniques. NCAAs such as AHA and regular ANL often require lengthy labeling periods of several hours for cell cultures, and even longer in rodents.^9^ As such, these techniques may be unable to detect the multiple temporally and spatially distinct changes in mRNA translation that are thought to underpin processes such as memory consolidation. By leveraging the inactivity of Opto-ANL, we are able to pretreat with the amino acid, allowing it to enter the cell prior to uncaging at a later time point. One of the many potential applications of being able to pretreat with Opto-ANL is to increase labeling at shorter time periods. By pretreating HEK293 cells with Opto-ANL prior to uncaging, we were able to observe a ≈50% increase in *de novo* proteomic labeling. In models where compound penetration is a greater concern, such as in brain slices, the increase in labeling enabled via pretreatment is likely even greater.^35^

Through the use of a NPOCC photocage, we were able to ensure that the uncaging of Opto-ANL can be promoted with very mild Uva light irradiation and within a short time frame and without noticeable background hydrolysis (Figure 2). This ease of uncaging not only increases the temporal resolution of our labeling technique, but also reduces the risk of significant physiological or morphological changes being caused via irradiation. This feature makes Opto-ANL ideal for use in mammalian cell cultures, tissue slices, and even animal models where UV light can be delivered.^36^ To further improve tissue penetration, red-shifted photocages (e.g., coumarin, BODIPY, Cyanine-based) could be employed instead of NPOCC. This would allow for more sophisticated in vivo applications, but would make the handling of the compound itself more challenging as it would become sensitive to ambient light.

Another advantage of our compound is that Opto-ANL combines both photopharmacology with genetic methods to enable cell-type labeling of the *de novo* proteome. One of the greatest limitations of photopharmacology when compared to optogenetic methods has been a lack of cell type-specificity. Despite tremendous advances in light delivery (e.g., redshifting,^37^ optoelectronics,^38^ and two photon imaging^39^), certain biological environments such as the brain remain too complex for light to be able to precisely differentiate between cell types. Approaches combining genetic manipulations and photopharmacology, such as tethered photopharmacology, have been largely successful at controlling biological machinery with spatial, temporal and cell-type specificity, and even making significant discoveries in neuroscience.^40−43^ Our technique also successfully leverages these two approaches, being able to selectively label the *de novo* proteome of hippocampal excitatory neurons in brain slices.

It is unclear whether Opto-ANL is able to cross the blood–brain barrier; however, the additional spatial and temporal control provided by our technique enables many exciting possibilities when studying cell-type specific changes in the *de novo* proteome during complex rodent behavior. Using cannulation, Opto-ANL could be delivered to specific brain regions for hours prior to uncaging with UVa light, not only greatly increasing labeling over short time periods, but also ensuring a constant supply of Opto-ANL throughout the behavioral task. This would also allow researchers to uncouple the delivery of the labeling compound with the behavior task, reducing the effects of animal stress on mRNA translation. Furthermore, by combining the cell-type specificity of ANL with photopharmacology, Opto-ANL could be used to examine local protein synthesis. This could be achieved by restricting UVa irradiation to anatomically distinct regions of the brain, allowing for Opto-ANL to only be uncaged in axons or dendrites. Lastly, Opto-ANL could be used in combination with photocaged versions of other previously described cell-type specific NCAAs. By utilizing photocages sensitive to different wavelengths of light, this approach would allow for either the simultaneous examination of *de novo* proteomic changes across multiple cell types, or to examine multiple temporal windows of protein synthesis within the same cells.

Overall, Opto-ANL can be used as a reliable tool for light-activatable and cell-type specific proteomic labeling of complex cells such as neurons. Observing changes in the *de novo* proteome through proteomic labeling is an essential tool for understanding complex biological processes, especially in the context of memory formation. The features of Opto-ANL are well matched to the challenges in studying *de novo* translation within specific cell populations and limited time frames. We believe that Opto-ANL will empower the development of more complex ANL-based assays and generate new insights into the temporal control of mRNA translation. More broadly, our study also highlights how photopharmacology can be used to elevate pre-existing techniques in molecular biology and improve their ability to explore complex and intricate biological phenomenon.

## Methods

### Chemical Synthesis

General information, experimental procedures, and characterization are summarized in the Supporting Information.

### Absorbance Standard Curve

Opto-ANL was dissolved in known concentrations in a solvent mixture of 1:1 CH_3_CN and H_2_O. Samples were submitted to high-pressure LCMS (see Supporting Information) and the resulting integration from the corresponding absorbance peak as detected by the diode array spectrophotometer at 265 nm was used to draw a standard ladder.

### Uncaging Kinetics

A 100 μM solution of Opto-ANL in a solvent mixture of 1:1 CH_3_CN and H_2_O was subjected to UV light irradiation (370 nm, 10 mW/cm^2^) using a PR160L-370 nm Gen 2 Kessil lamp. Time points were collected 60 s for a total of 480 s. The resulting photolysis products were analyzed by high-pressure LCMS. The integration from the absorbance peak at 265 nm corresponding to Opto-ANL for each time point was recorded and fit into the standard curve to determine the remaining concentration of Opto-ANL.

### NMR Analysis of Uncaged Products

To confirm the uncaging of Opto-ANL, the compound was dissolved in deuterated MeOH and placed in a quartz NMR tube and irradiated with UV light (370 nm, 10 mW/cm^2^) for 5 min. ^1^H NMR spectrum of the sample was acquired and compared to the spectra of Opto-ANL.

### Cell Culture

HEK293 cells were cultured in phenol-red free Dulbecco’s modified Eagle’s medium (Thermo Fisher, 21,063,029) supplemented with 10% FBS and 50 U/mL penicillin/streptomycin at 37 °C in a 5% CO_2_ saturated humidity incubator. Cells were plated onto coverslips pretreated with 0.01% poly l-ornithine solution (Millipore-Sigma, A-004-C). Cells were then transfected with pMars-L274G (Addgene, 63,177) using lipofectamine LTX (Thermo Fisher, 15,338,100), as per the manufacturer’s instructions.

### De Novo Proteomic Labeling with Opto-ANL in Cell Culture

All experiments utilizing Opto-ANL were performed under red-light to avoid accidental uncaging. To label newly synthesized proteins, cells and mouse brain slices were treated with either 1 mM Opto-ANL or ANL prior to uncaging. To uncage Opto-ANL in cell-culture experiments, the cell DISCO system as previously described in the literature (citation) was used with constant irradiation from 370 nm, 4.0–6.0 mW light emitting diodes purchased from Roithner Lasetechnik (XSL-370–5E). Following uncaging, samples were incubated in the dark to protect from UV light. For experiments where protein synthesis was inhibited, 50 μg/mL CHX (Millipore-Sigma, 239,763) was added to the samples 5 min prior to treatment with Opto-ANL or ANL. Following *de novo* proteomic labeling with Opto-ANL, samples were fixed using 4% paraformaldehyde for 20 min.

### FUNCAT Western Blot Analysis

Proteins were extracted in equal volumes of 1X radioimmunoprecipitation assay (RIPA) buffer (Cell Signaling, 9806) with Halt protein inhibitor cocktail (ThermoFisher, 78,438) and 1 mM phenylmethylsulfonyl fluoride (ThermoFisher, 36,978). Newly synthesized proteins were then fluorescently labeled with IRDye800CW-Alkyne (LiCOR, 929–60,002) using Click-&-Go Protein Reaction Buffer Kit (Vector Laboratories, CCT-1262) as per the manufacturer instructions. Proteins were then precipitated using methanol chloroform precipitation,^44^ before being resuspended in 5% SDS sample buffer. Samples were then denatured via boiling in 1 × Laemmli buffer, before being separated via SDS–PAGE and transferred to a PVDF membrane using the iBlot semidry transfer system (Invitrogen, IB2100). For the detection of mCherry, membranes were first blocked with Odyssey tris-buffered saline blocking buffer (LI-COR, 927–50,000), before being incubated overnight at 4 °C with rat anti-mCherry antibody (ThermoFisher, M11217, 1:1000). After washing, membranes were stained with IRDye680 antirat IgG (LI-COR, 926–68,076, 1:10,000) before being imaged using a LI-COR Odyssey M scanner. The amount of FUNCAT signal was quantified using the LI-COR Emperia Studio software, with the total protein stain REVERT (LI-COR, 926–10,011) used for normalization.

### Cytotoxicity Assay

Cells were treated with 1 mM Opto-ANL or ANL prior to being treated with our UV uncaging paradigm. Four hours later, cells were incubated with CytoPainter Fixable Cell Viability (abcam, ab176745; 1:100) for 30 min at 37 °C in a 5% CO_2_ in a saturated humidity incubator. Cells were then fixed with 4% PFA, before being stained using DAPIa mounted in ProLong Gold mounting media. As a positive control, cells were incubated at 60 °C for 10 min prior to the addition of the CytoPainter dye.

### De Novo Proteomic Labeling with Opto-ANL in Mouse Brain Slices

R26-MetRS X Camk2a-Cre mice, along with WT littermates were maintained in the Transgenic Mouse Facility of New York University in accordance with the US National Institutes of Health Guide for Care and Use of Laboratory Animals. Brain slices were prepared from these mice as previously described,^45^ with minor modification. Briefly, 400 μm-thick brain slices containing the hippocampus were sectioned using a vibratome and allowed to recover in artificial cerebral spinal fluid at 32 °C for 1 h. Samples were then treated with Opto-ANL, with a PR160L-370 nm Gen 2 Kessil lamp being used to induce uncaging. Following this, samples were fixed overnight in 4% paraformaldehyde before being resectioned at 40 μm.

### FUNCAT and Immuno-Staining

Opto-ANL labeled proteins were detected as previously described,^46^ with minor modifications. Briefly, fixed samples were blocked for 1 h at RT under constant agitation in 5% bovine serum album, 5% normal goat serum, and 0.5% triton-x in phosphate buffered saline. Newly synthesized proteins were then visualized with Alexa 647 alkyne using the Click-iT Cell Reaction Buffer Kit (ThermoFisher, C10269) as per the manufacturer’s instructions. Cells expressing L274G-MetRS were detected using a rat anti-mCherry antibody (ThermoFisher, M11217, 1:500). Anti-β-actin (Abcam, ab8226, 1:500) and anti-GFAP (EnCor Biotechnology, AB_2109953, 1:500) antibodies were used to detect cells and astrocytes, respectively. Cell nuclei were stained using DAPI and samples were mounted in ProLong Gold mounting media (ThermoFisher, P10144).

### Imaging and Image Analysis

Fifteen μm thick *Z*-stack images were taken using a Leica SP8 Confocal microscope with maximum intensity projection being created in ImageJ. Image analysis was performed blinded with mCherry or β-actin staining being used to create a mask as appropriate. Mean gray value was measured within this mask for each image. No significant difference was detected in the areas of these masks across groups. Each data point represents the average of at least three technical replicates.

### Statistical Analysis

Statistical analysis was performed in GraphPad Prism 10.1.2 software, using one-way ANOVA with Tukey’s multiple comparison test (MCT), as appropriate. All values are given as mean ± standard error of the mean. Significance was defined as *p < 0.05, **p < 0.01, ***p < 0.001, ****p < 0.0001.

## Supporting Information Available

Supporting Information figures, additional experimental details, synthetic procedures, and characterization data for all compounds (PDF)
