# Supplementary material for: Light-Activatable, Cell-Type Specific Labeling of the Nascent Proteome
Source: ACS Chem Neurosci. 2024 Sep 23;15(19):3473–81. doi: 10.1021/acschemneuro.4c00274 (PMC11450754; doi:10.1021/acschemneuro.4c00274)
Supplement: Supplementary file 1 — cn4c00274_si_001.pdf [file cn4c00274_si_001.pdf]

Supporting Information

for

Light-activatable, cell-type specific labeling of the nascent proteome

H.T. Evans[‡,^], T. Ko[#,^], M.M. Oliveira[‡], A. Yu[‡], S.V. Kalavai[‡], E.N. Golhan[‡], A. Polavarapu[‡], E.Balamoti[‡], V.Wu[‡], E. Klann[*,‡,^], D. Trauner[*,#,^]

[^] Designates equal contribution of authors

[‡] Center for Neural Science, New York University, New York, New York, 10003, United States

[#] Department of Chemistry, University of Pennsylvania, Philadelphia, Pennsylvania, 19104, United States

[*] ek65@nyu.edu, dtrauner@upenn.edu

Table of Contents

Supplemental Figures S3

General Information and Protocols S5

Synthetic Procedures and Characterization S7

NMR spectra S9

References S11

Supplemental Figures

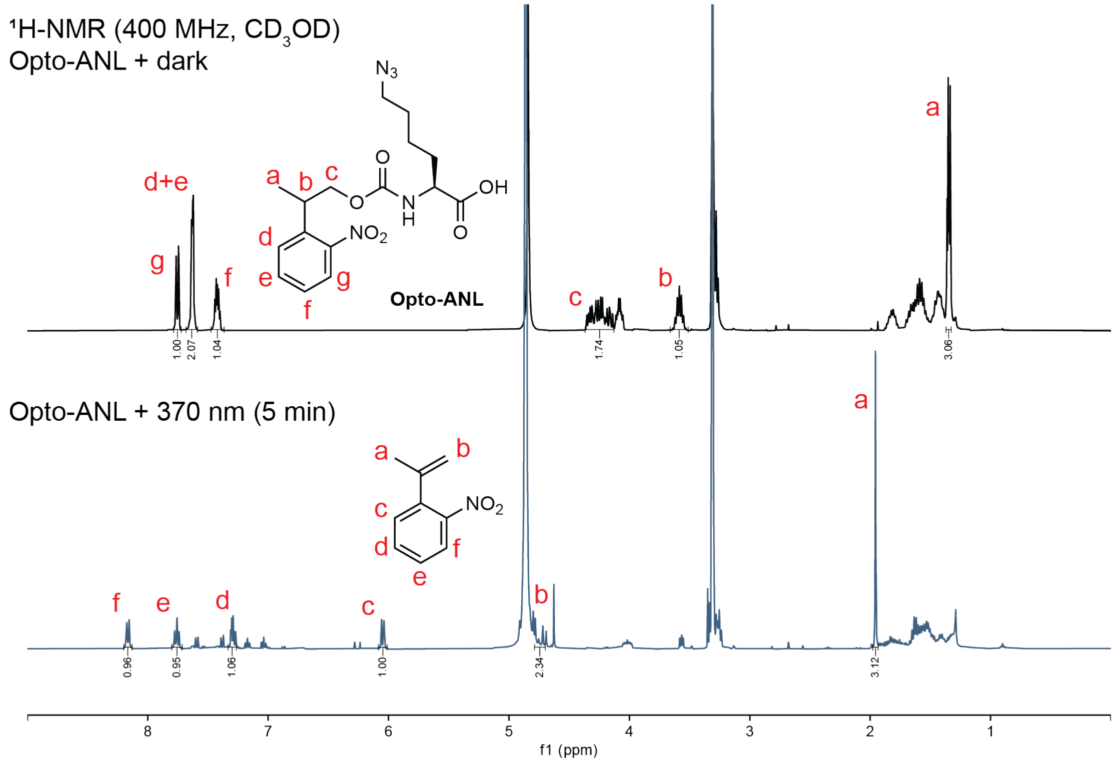

<u>Supplementary Fig 1.</u> Uncaging of Opto-ANL as monitored by 1H-NMR in deuterated MeOH. Sample of Opto-ANL was irradiated for 5 min with 370 nm light (10mW/cm^2) and the peaks corresponding to the styrene degradation product were identified.

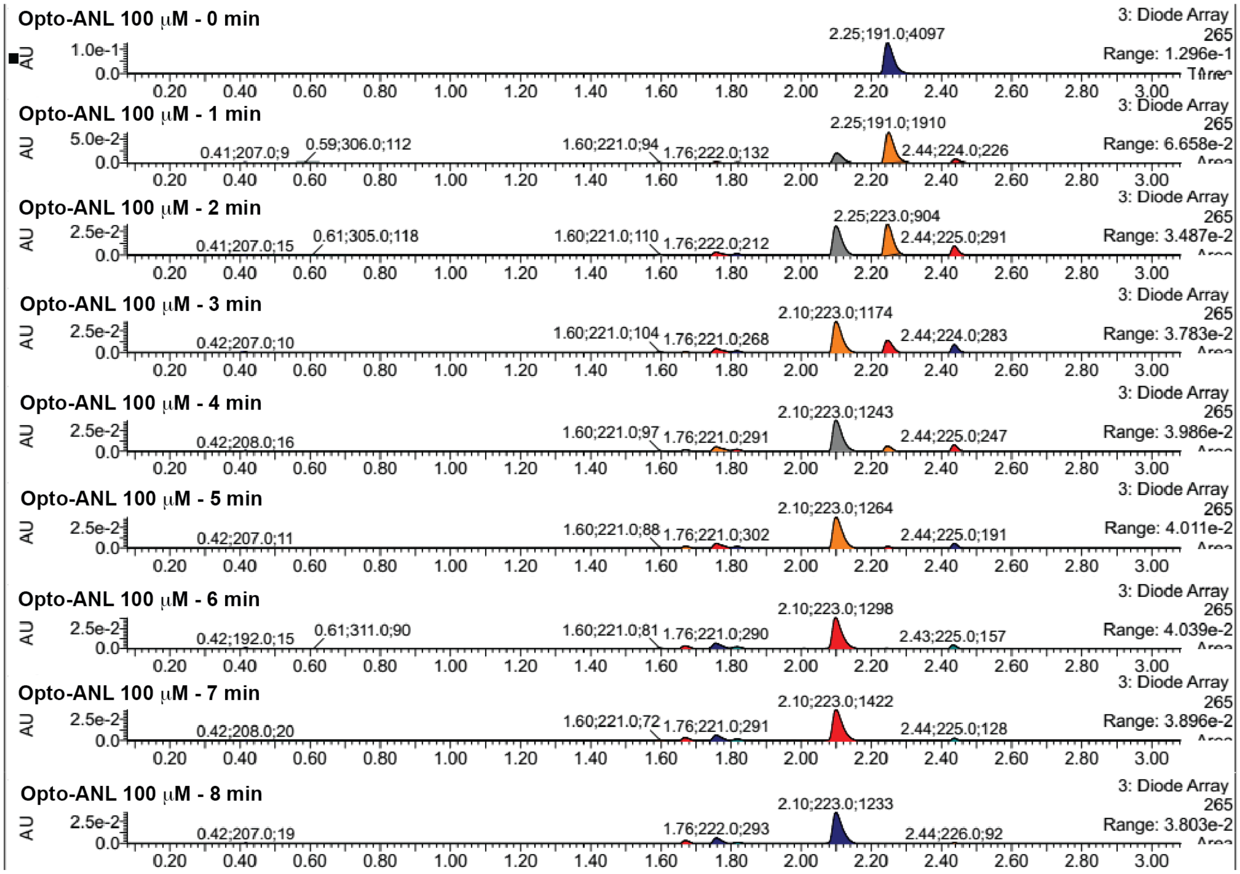

<u>Supplementary Fig 2.</u> Uncaging of Opto-ANL (100 µM) as monitored by high-pressure LCMS in 1:1 CH_3CN and H_2O. Sample of Opto-ANL was irradiated during minute intervals with 370 nm light (10mW/cm^2) and the chromatograms at 265 nm absorbance were recorded.

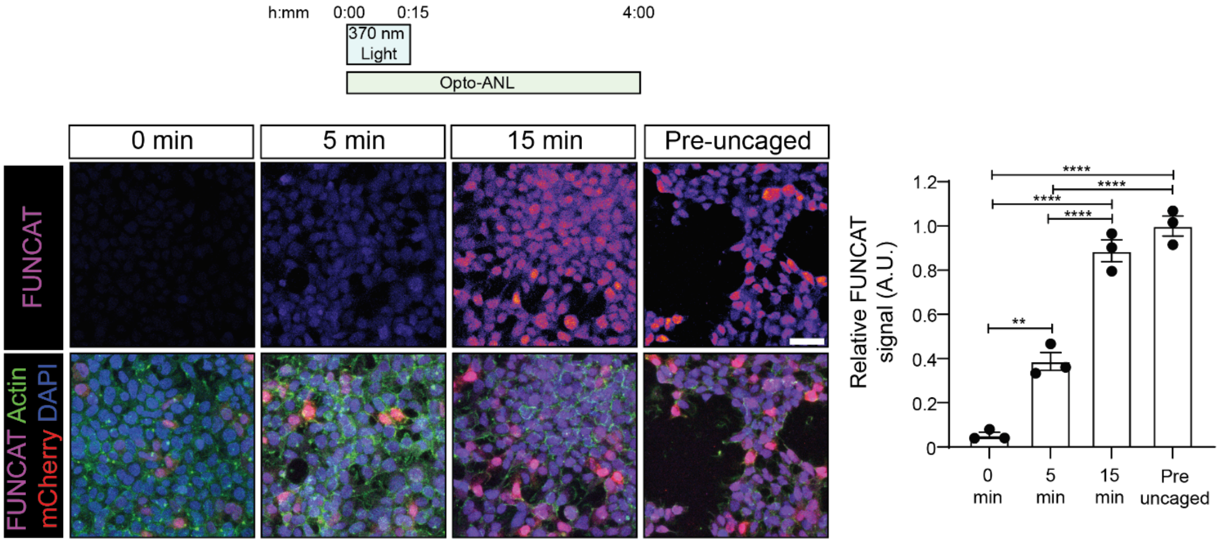

Supplementary Fig 3. 15 minutes of low intensity UV irradiation is sufficient to induce complete uncaging of Opto-ANL in HEK293 cells. Cells were treated with 1mM Opto-ANL before being irradiated for varying times with 370 nm light. Cells were fixed 4 hours after Opto-ANL treatment. As a positive control, cells were treated with pre-uncaged Opto-ANL. Cells irradiated with UV light for 15 minutes showed similar FUNCAT signal to cells treated with pre-uncaged Opto-ANL (one-way ANOVA, Tukey's MCT, n=3 experiments, 4 technical replicates per experiment, **=p≤0.01, ****=p≤0.0001, error bars= S.E.M).

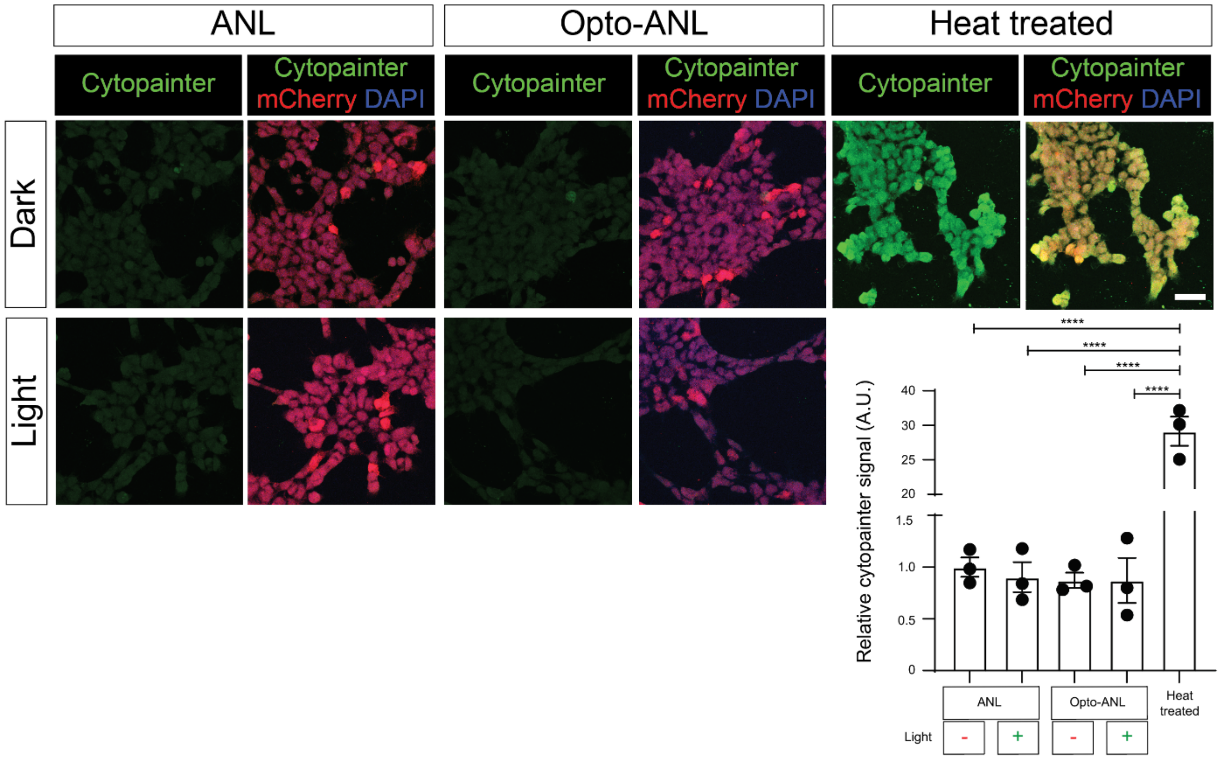

Supplementary Fig 4. Opto-ANL treatment and UV caging does not induce cytotoxicity. Cells were treated with ANL or Opto-ANL prior to 15 minutes of low intensity UV irradiation. 4 hours later, cells were incubated with CytoPainter for 30 minutes. Opto-ANL+UV treated cells showed no difference in CytoPainter fluorescence compared to cells treated with ANL in the absence of UV light, indicating a lack of cytotoxicity. All groups showed significantly lower Cyto-Painter fluorescence compared to cells which underwent heat shock prior to incubation of CytoPainter (one-way ANOVA, Tukey's MCT, n=3 experiments, 4 technical replicates per experiment, **=p≤0.01, ****=p≤0.0001, error bars= S.E.M).

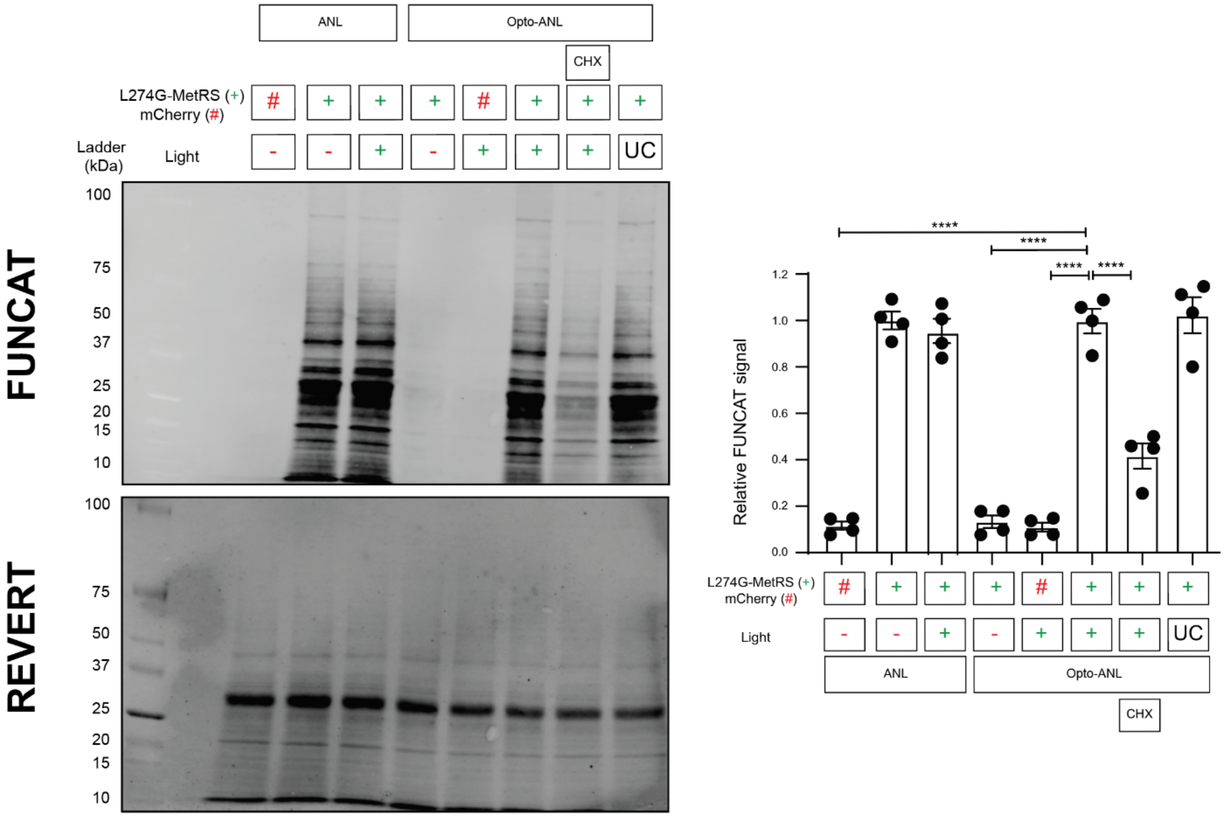

Supplementary Fig 5. Western blot analysis confirm that Opto-ANL enables light-activatable, L274G-MetRS dependent labelling of the de novo proteome. HEK293 cells were transfected with L274G-MetRS-mCherry or an mCherry plasmid, before being treated with Opto-ANL and then irradiated with mild UV light for 15 minutes prior to analysis via FUNCAT WB. FUNCAT signal was ablated in the absence of light or L274G-MetRS, and is also reduced by the addition of the protein synthesis inhibitor, CHX. FUNCAT signal was similar in Opto-ANL+Light samples compared to ANL, ANL+Light samples, suggesting that our irradiation paradigm is sufficient to induce complete uncaging of Opto-ANL, while not altering global protein synthesis levels. (one-way ANOVA, Tukey's MCT, n=4 experiments, ****=p≤0.0001, error bars= S.E.M).

General Information and Protocols

General experimental conditions

All reactions were conducted with magnetic stirring at room temperature unless otherwise noted. No unexpected or unusually high safety hazards were encountered.

Chemicals

All chemicals were obtained from common vendors and used without further purification unless otherwise noted. All solvents were purchased with "certified ACS" or higher quality.

Chromatography

Retardation Factors (Rf) were determined by analytical thin-layer chromatography (TLC) performed on pre-coated glass plates from Millipore Sigma (TLC Silica Gel 60 Plates, 250 µm layer thickness, F254 fluorescence indicator), with visualization by exposure to ultraviolet light (254 nm). Column chromatography was performed with silica gel obtained from Millipore Sigma (Geduran® Si 60, 40 – 63 µm). Column chromatography was either performed manually or with an automated chromatography system (Teledyne Isco CombiFlash®).

Nuclear magnetic resonance (NMR) spectroscopy

Proton and carbon (^{1}H- and ^{13}C-) NMR spectra were recorded on Bruker NEO400 (400/100 MHz, with BBFO Cryoprobe™). All spectra were recorded at a temperature of 25 °C in 5 mm tubes in deuterated solvents purchased from Cambridge Isotope Laboratories, Inc. (chloroform-*d* or $CDCl_3$, 99.8% D; methanol-*d4* or MeOD-d_4, 99.8% D). For 1H-NMR spectra chemical shifts (δ) in parts per million (ppm) relative to tetramethylsilane (δ = 0 ppm) are reported using the residual protic solvent ($CHCl_3$ in $CDCl_3$: δ = 7.26 ppm, MeOD-d_3 in MeOD-d_4: δ = 3.31 ppm) as an internal reference. For ^{13}C-NMR spectra, chemical shifts in ppm relative to tetramethylsilane (δ = 0 ppm) are reported using the central resonance of the solvent signal ($CDCl_3$: δ = 77.16 ppm, MeOD-d_4: δ = 49.00 ppm) as an internal reference. The abbreviations used for multiplicities and descriptors are s = singlet, d = doublet, t = triplet, q = quartet or combinations thereof, m = multiplet and br = broad. NMR spectral data was analyzed with the program MestreNova.

Mass spectrometry (MS)

Liquid Chromatography-Mass Spectrometry (LCMS): Samples were measured on a Waters Acquity UPLC system equipped with a Waters diode array detector (DAD) detector (200-400 nm) and a Waters SQD single quadrupole mass analyzer with electrospray ionization. Elution was performed through an Acquity UPLC HSS C18 (1.7mm, 2.1 x 50 mm) column with a 30 seconds hold 95:5 (water:acetonitrile with 0.1% v/v formic acid), 2 minute gradient to 5:95, and 30 seconds hold.

High-resolution mass spectra (HRMS): Spectra were obtained with a Waters GCT Premier, time-of-flight, GCMS with electron ionization (EI), or an LCT Premier XE, time-of-flight, LCMS with electrospray ionization (ESI). Samples were taken up in a suitable solvent for analysis. The signals were mass measured against an internal lock mass reference of perfluorotributylamine (PFTBA) for EI-GCMS, and leucine enkephalin for ESI-LCMS. All reported data refers to positive ionization mode.

Synthetic Procedures and Characterization

2,5-dioxopyrrolidin-1-yl (2-(2-nitrophenyl)propyl) carbonate (**1**)

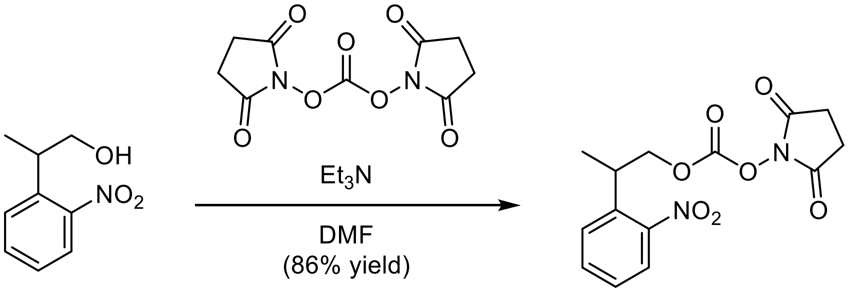

N,N′-Disuccinimidyl carbonate (0.424 g, 1.656 mmol, 1.5 eq.) under nitrogen atmosphere was dissolved in 4.8 ml of anhydrous DMF. The solution was sonicated until fully dissolved and added to a flask containing 2-(2-Nitrophenyl)propan-1-ol (0.2 g, 1.104 mmol, 1.0 eq.). Triethylamine (0.844 mL, 0.614 g, 6.071mmol, 5.5 eq.) was added dropwise and the solution was stirred overnight protected from ambient light. The stir bar was removed and Celite® added until the mixture has the consistency of damp sand. The reaction flask was dried under reduced pressure and the resulting powder directly purified by column chromatography (0 to 90% gradient of ethyl acetate in hexanes) to afford the title compound (0.304 g, 0.943 mmol, 86%) as a clear viscous oil.

$\mathbf{R_f}$ = 0.4 (50% ethyl acetate in hexanes)

^{1}H-NMR (400 MHz, $CDCl_3$) δ 7.82 (dd, J = 8.1, 1.4 Hz, 1H), 7.61 (td, J = 7.6, 1.4 Hz, 1H), 7.49 (dd, J = 8.0, 1.4 Hz, 1H), 7.41 (ddd, J = 8.1, 7.3, 1.4 Hz, 1H), 4.57 – 4.46 (m, 2H), 3.79 (h, J = 6.7 Hz, 1H), 2.80 (s, 4H), 1.43 (d, J = 7.0 Hz, 3H).

^{13}C-NMR (100 MHz, $CDCl_3$) δ 168.64, 151.50, 150.07, 135.92, 133.16, 128.60, 128.06, 124.62, 74.48, 33.44, 25.51, 17.56, 7.26, 1.59.

**LCMS** (ESI):	t_{ret} = 2.11 min	323.340 m/z $[M+H]^+$.
**HRMS** (ESI):	calc. for $C_{14}H_{14}N_2O_7Na^+$:	345.0699 m/z $[M+Na]^+$.
	found:	345.0699 m/z $[M+Na]^+$.

The spectroscopic data agrees with previously reported values.[1]

Opto-ANL

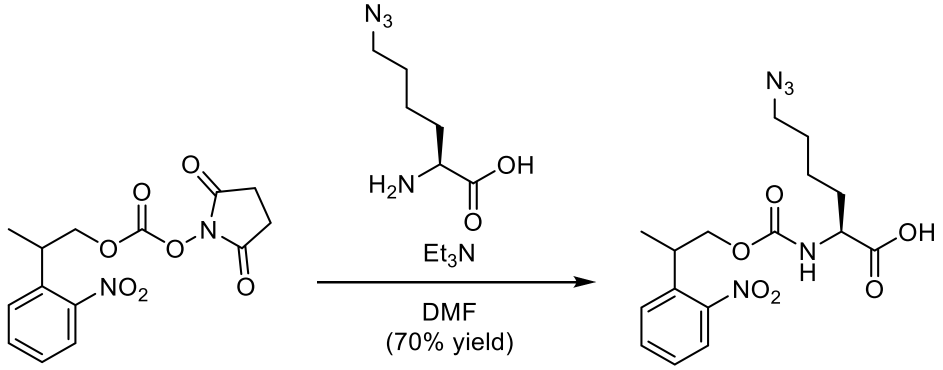

To L-azidonorleucine hydrochloride (20.0 mg, 0.096 mmol, 1.0 eq.), a solution of 2,5-dioxopyrrolidin-1-yl (2-(2-nitrophenyl)propyl) carbonate (**1**, 35.9 mg, 0.112 mmol, 1.16 eq.) in 1.7 ml of DMF was added under nitrogen atmosphere. The mixture was stirred until all solids were dissolved and triethylamine (87 µl, 0.627 mmol, 6.5 eq) was added dropwise. The solution was stirred overnight at room temperature and concentrated under reduced pressure until most of the DMF had evaporated. The resulting residue was dissolved in 2.5 ml of ethyl acetate and 2.5 ml of 10% LiCl aqueous solution. Subsequently, the aqueous layer was extracted (2 x 2.5 ml) with ethyl acetate. The organic layers were combined, washed with saturated aqueous sodium chloride (2.5 mL) and dried over sodium sulfate. The solvent was removed under reduced pressure and the residue purified by column chromatography (0 to 100% gradient of ethyl acetate with 1% acetic acid in dichloromethane) to afford the title compound (25.3 mg, 0.067 mmol, 70%) as a yellow viscous oil.

R_f = 0.43 (50% ethyl acetate and 1% acetic acid in dichloromethane)

^{1}H NMR (400 MHz, MeOD-*d*$_4$) δ 7.75 (d, J = 8.1 Hz, 1H), 7.67 – 7.60 (m, 2H), 7.47 – 7.37 (m, 1H), 4.37 – 4.13 (m, 2H), 4.08 (td, J = 9.2, 4.7 Hz, 1H), 3.66 – 3.51 (m, 1H), 3.30 – 3.25 (m, 2H), 1.82 (dq, J = 13.4, 6.9 Hz, 1H), 1.72 – 1.62 (m, 1H), 1.57 (tt, J = 13.4, 6.8 Hz, 2H), 1.50 – 1.37 (m, 2H), 1.35 (dd, J = 7.0, 3.7 Hz, 3H).

^{13}C-NMR (100 MHz, MeOD-*d*$_4$) δ 175.71, 158.43, 152.07, 138.48, 138.37, 133.76, 133.74, 129.64, 128.61, 124.92, 69.57, 55.02, 52.22, 34.96, 32.21, 29.38, 24.07, 18.21.

**LCMS** (ESI):	t_{ret} = 2.19 min	380.473 m/z $[M+H]^+$.
**HRMS** (ESI):	calc. for $C_{16}H_{22}N_5O_6^+$:	380.1570 m/z $[M+H]^+$.
	found:	380.1569 m/z $[M+H]^+$.

^{1}H-NMR (400 MHz, $CDCl_3$) – **1**

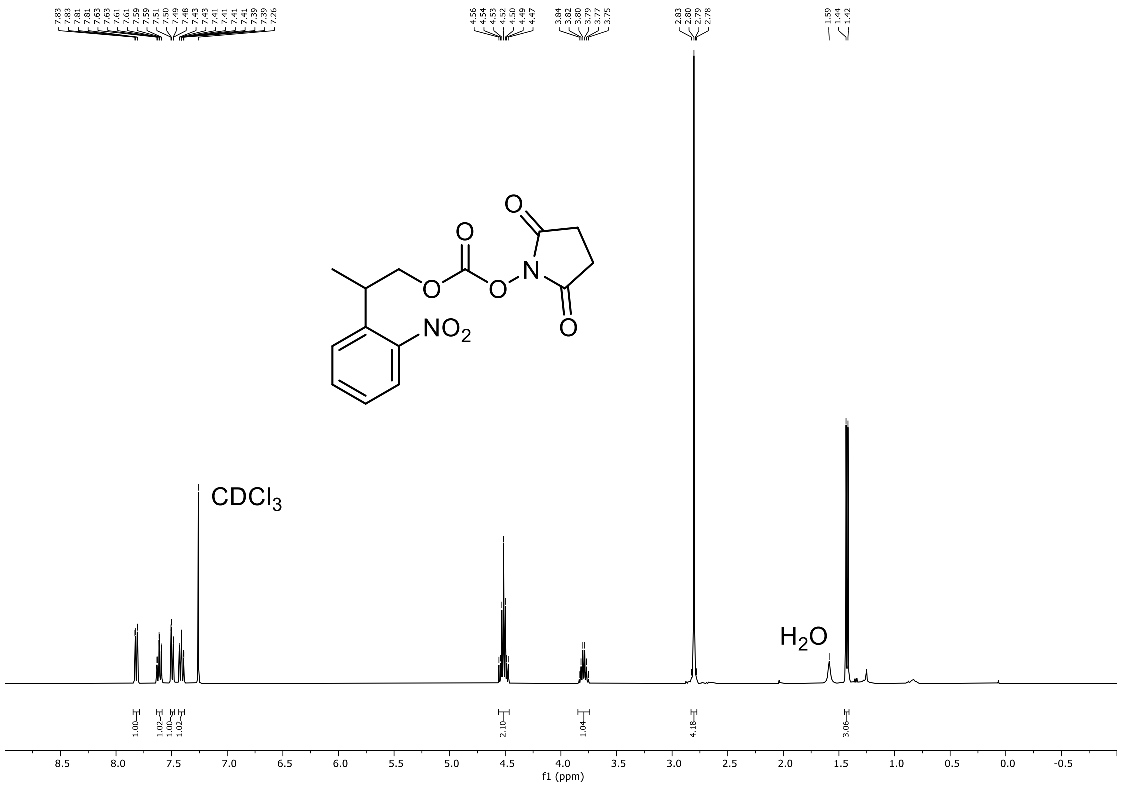

^{13}C-NMR (100 MHz, $CDCl_3$) – **1**

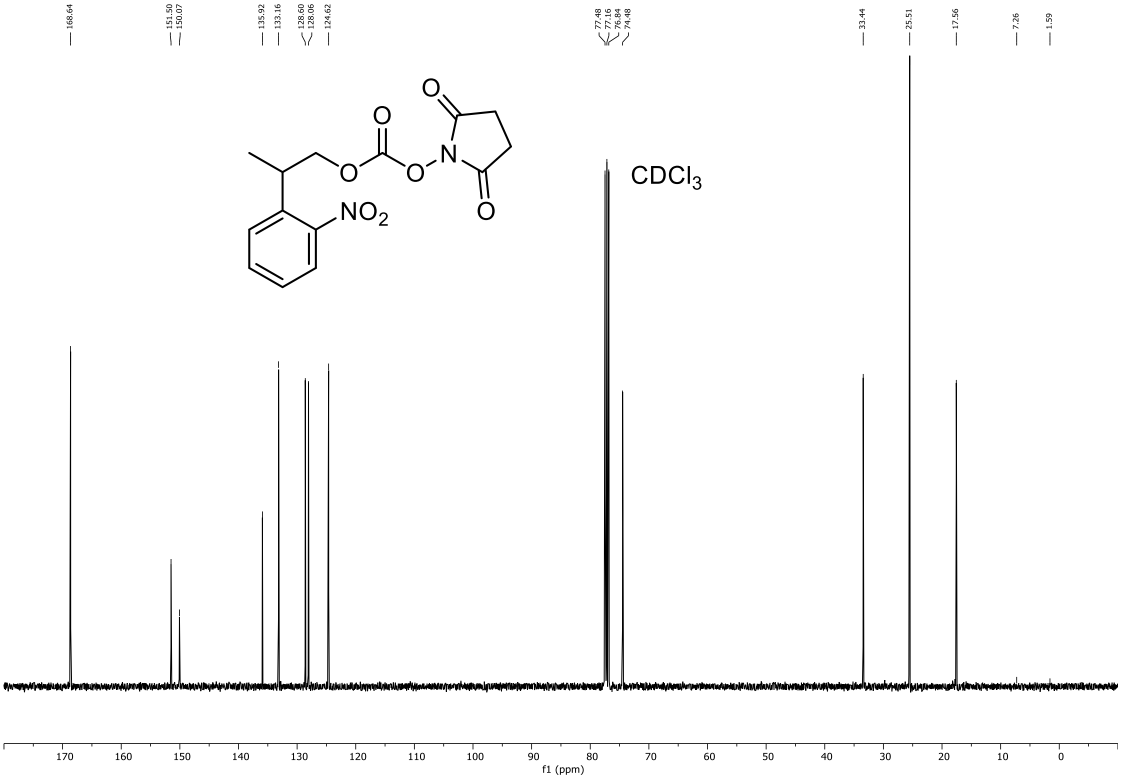

^{1}H-NMR (400 MHz, CD_3OD) – **Opto-ANL**

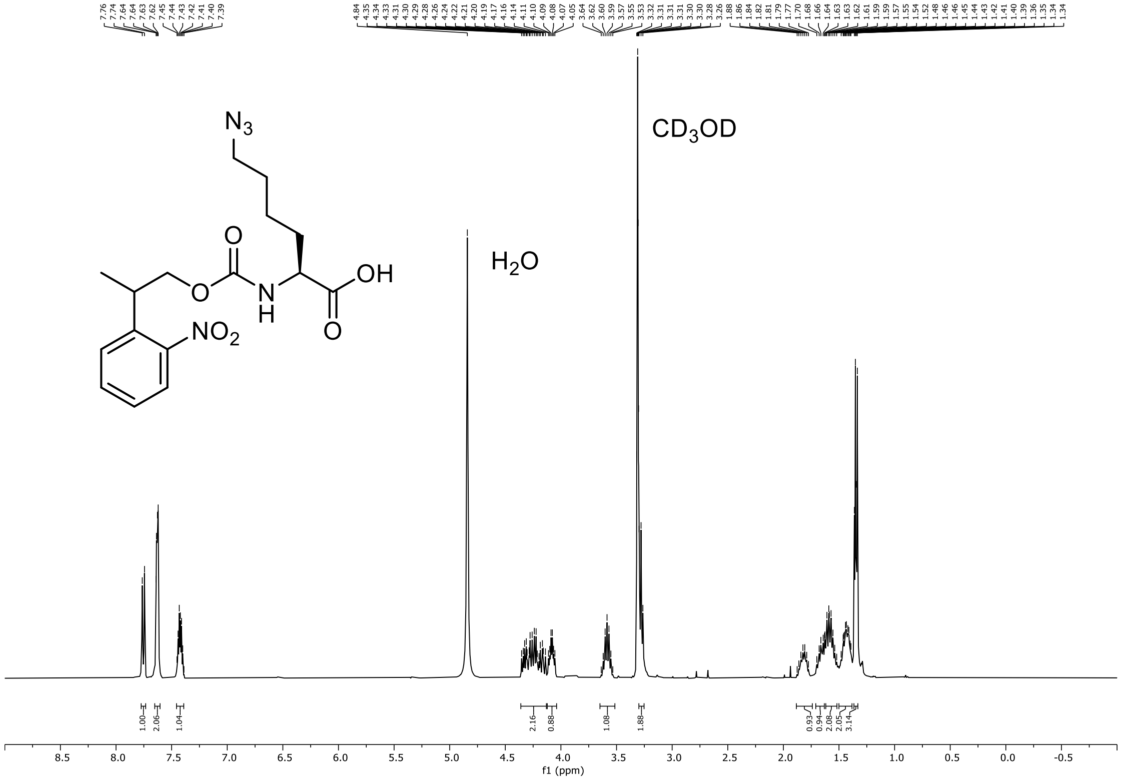

^{13}C-NMR (100 MHz, CD_3OD) – **Opto-ANL**

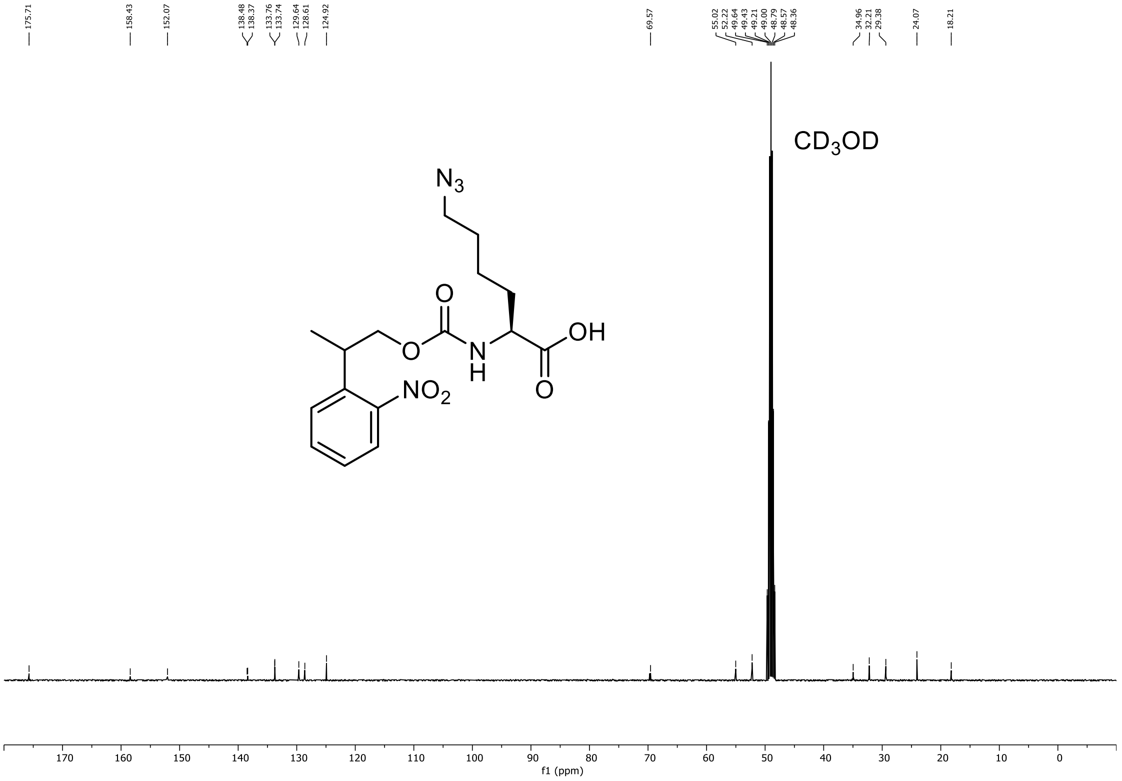

References

(1) DeForest, C. A.; Tirrell, D. A. A Photoreversible Protein-Patterning Approach for Guiding Stem Cell Fate in Three-Dimensional Gels *Nature Materials* **2015**, *14*, 523-531.
